# Systemic Delivery of Bone Marrow Mesenchymal Stem Cells for In Situ Intervertebral Disc Regeneration

**DOI:** 10.5966/sctm.2016-0033

**Published:** 2016-10-11

**Authors:** Carla Cunha, Catarina R. Almeida, Maria Inês Almeida, Andreia M. Silva, Maria Molinos, Sofia Lamas, Catarina L. Pereira, Graciosa Q. Teixeira, António T. Monteiro, Susana G. Santos, Raquel M. Gonçalves, Mário A. Barbosa

**Affiliations:** ^1^ i3S‐Instituto de Investigação e Inovação em Saúde, Universidade do Porto, Porto, Portugal; ^2^ INEB‐Instituto de Engenharia Biomédica, Universidade do Porto, Porto, Portugal; ^3^ Department of Medical Sciences and Institute for Biomedicine, University of Aveiro, Aveiro, Portugal; ^4^ ICBAS‐Instituto de Ciências Biomédicas Abel Salazar, Universidade do Porto, Porto, Portugal; ^5^ IBMC‐Instituto de Biologia Molecular e Celular, Universidade do Porto, Porto, Portugal; ^6^ Research Centre on Biodiversity and Genetic Resources, CIBIO‐InBIO Associate Laboratory, Vairão, Portugal

**Keywords:** Intravenous transplantation, Cell therapy, Herniation, Immunomodulation, Paracrine

## Abstract

Cell therapies for intervertebral disc (IVD) regeneration presently rely on transplantation of IVD cells or stem cells directly to the lesion site. Still, the harsh IVD environment, with low irrigation and high mechanical stress, challenges cell administration and survival. In this study, we addressed systemic transplantation of allogeneic bone marrow mesenchymal stem cells (MSCs) intravenously into a rat IVD lesion model, exploring tissue regeneration via cell signaling to the lesion site. MSC transplantation was performed 24 hours after injury, in parallel with dermal fibroblasts as a control; 2 weeks after transplantation, animals were killed. Disc height index and histological grading score indicated less degeneration for the MSC‐transplanted group, with no significant changes in extracellular matrix composition. Remarkably, MSC transplantation resulted in local downregulation of the hypoxia responsive GLUT‐1 and in significantly less herniation, with higher amounts of Pax5+ B lymphocytes and no alterations in CD68+ macrophages within the hernia. The systemic immune response was analyzed in the blood, draining lymph nodes, and spleen by flow cytometry and in the plasma by cytokine array. Results suggest an immunoregulatory effect in the MSC‐transplanted animals compared with control groups, with an increase in MHC class II+ and CD4+ cells, and also upregulation of the cytokines IL‐2, IL‐4, IL‐6, and IL‐10, and downregulation of the cytokines IL‐13 and TNF‐α. Overall, our results indicate a beneficial effect of systemically transplanted MSCs on in situ IVD regeneration and highlight the complex interplay between stromal cells and cells of the immune system in achieving successful tissue regeneration. Stem Cells Translational Medicine *2017;6:1029–1039*

**Significance Statement**

This study assesses the effects of bone marrow‐derived mesenchymal stem cells (MSCs) transplanted systemically for intervertebral (IVD) regeneration into a rat IVD lesion model. It demonstrates systemic MSC transplantation is effective in inhibiting disc degeneration and herniation in situ. Moreover, a systemic immunomodulatory effect of MSC transplantation was shown, demonstrating the involvement of the immune system in vivo in the local effect observed in the IVD and hernia. This study highlights the complex interplay between stromal cells and cells of the immune system in achieving successful IVD tissue regeneration. This is considered to be the first study in which the systemic immune response was assessed together with the effect of MSC transplantation on IVD tissue regeneration.

## Introduction

Intervertebral disc (IVD) degeneration and herniation, with its often associated proinflammatory environment, is one of the most common causes of low back pain [1]. The IVD is a complex tissue, noninnervated and minimally irrigated, composed by a central proteoglycan and collagen type II‐rich nucleus pulposus (NP) and by the peripheral fibrous, collagen type I‐rich annulus fibrosus (AF) [2]. Present treatments focus on symptomatic pain relief or surgery but fail to address the mechanisms behind IVD pathophysiology. Different tissue engineering approaches have been developed to address IVD degeneration, including gene transfer, molecular therapy, and cell therapy, with and without biomaterial‐assisted delivery [3]. Main approaches for cell therapy rely on transplantation of IVD‐derived cells, transplantation of exogenous cells such as stem cells, or transplantation of factors that might stimulate tissue regeneration through the activation of endogenous cells [4].

Bone marrow mesenchymal stem cells (MSCs), in particular, have important roles in repairing damaged tissue, given their broad tissue distribution, multipotent differentiation capacity, and nonimmunogenicity [5], which make MSCs very appealing for IVD regeneration. MSCs were shown to delay the IVD progressive degenerative process and, in some cases, to induce regeneration features [6]. In vitro, these cells were able to differentiate into NP‐like cells and to induce glycosaminoglycans and collagen type II production when cocultured with IVD cells [7, 8]. In vivo, MSCs transplanted to the IVD have been shown to increase matrix production, disc hydration, disc height, and glycosaminoglycans production [9, 10, 11]. Furthermore, it is well established that MSCs have immunomodulatory properties, which depend on the microenvironment encountered [12]. MSC transplantation for IVD degeneration is already in phase II clinical trials: A study used bone marrow MSC transplantation with hyaluronic acid as carrier to treat lumbar disc degeneration [13]. Although results from clinical trials appear promising, outcomes have been insufficient to date and failed to show an MSC cause‐and‐effect improvement.

These studies were based on local transplantation of MSCs to the IVD, relying on MSCs’ capability to give rise to different lineages and replace the damaged tissue. However, the harsh microenvironment of the degenerated IVD, including hypoxia, low nutrition, acidic pH, and high mechanical loading, pose difficulties for MSC survival [14]. In fact, the effect of MSC transplantation in the proinflammatory/degenerated IVD microenvironment still remains to be completely understood [15]. Also, MSC administration to the IVD is impaired by its high mechanical loading, leading to possible cell leaking and possible osteophyte formation [16]. Moreover, it requires a needle puncture, which may cause disc damage [17]. More recently, some studies have addressed MSCs’ role in what is usually referred to as endogenous mechanisms of IVD regeneration, especially through stem or progenitor cell homing [18, 19]. This approach is supported by the knowledge that stem cell recruitment in musculoskeletal injury is a natural healing response [20] and that different inflammatory mediators and cells stimulate MSC recruitment [21, 22]. MSC recruitment has been shown to occur in response to IVD degenerative conditions in a bovine whole‐organ culture [23]. Using the same model, our group has demonstrated that MSC recruitment to the degenerated IVD is potentiated by the chemoattractant SDF‐1 using a hyaluronan‐based delivery system [24]. Recently, the same effect has been demonstrated in vivo: Bone marrow MSC recruitment occurred in the mouse IVD 12 weeks after intravenous administration and was shown to be related to the degree of IVD degeneration [25].

In addition to trafficking and homing to the lesion site, MSCs also participate in the repair process via the so‐called cell empowerment. MSCs produce large quantities of growth factors, which, in situ, stimulate endothelial cells to angiogenesis and endothelial repair, fibroblasts to ECM remodeling, and tissue progenitor cells for differentiation. MSC paracrine communication with the inflammatory microenvironment is an essential part of this process. Some reports have already demonstrated that MSCs are able to communicate bidirectionally with IVD cells, in vitro, through secretion of bioactive factors [26], indicating MSCs’ immunomodulatory and trophic effects are potentially able to modulate and guide the IVD repair process.

In this study, we propose to transplant bone marrow MSCs systemically into a rat model of IVD lesion. Our aim is to explore the role of MSCs in inhibiting degeneration in situ and to understand the contribution of the MSC‐immune system communication to this process.

## Materials and Methods

### Animal Experimentation

The inbred Lewis (LEW/Crl) strain of rats was used to reduce possible allogeneic cell rejection. A total of 30 male rats (*n* = 6 per group), 2 months old, were used. The IVD lesion was performed by caudal needle puncture, as described previously [27]. Briefly, a percutaneous 21‐gauge needle puncture was performed into 3 consecutive coccygeal IVDs (Co5/6, Co6/7, Co7/8), with the help of radiography for disc identification. The naïve group refers to littermates that were healthy and unlesioned. Experiments were carried out at the Instituto de Biologia Molecular e Celular.Instituto de Engenharia Biomédica (IBMC.INEB) animal house in accordance with European Legislation on Animal Experimentation through Directive 2010/63/UE and approved by the IBMC.INEB Animal Ethics Committee and by Direcção Geral de Alimentação e Veterinária through license no. 3773/2015‐02‐09.

### Cell Isolation and Transplantation

Rat bone marrow MSCs (rMSCs) were isolated from the femoral and tibial bone marrow. rMSCs were cultured in minimum essential medium alpha modification with 10% MSC‐qualified fetal bovine serum (FBS; Thermo Fisher Scientific Life Sciences, Waltham, MA, http://www.thermofisher.com) and expanded up to P5. rMSCs were characterized for their phenotypic profile as CD29+, CD90+, CD45− and for their differentiation potential into osteogenic, chondrogenic, and adipogenic lineages [21] (supplemental online Fig. 1).

**Figure 1:**
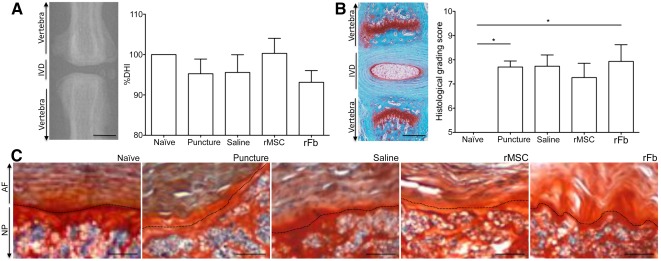
Radiograph, bar charts of %DHI and histological grading score, and micrographs of stained tissue **(A–C)**. DHI is calculated by measurement of disc height seen in digital radiographs. **(A):** All 3 lesioned discs in each animal were quantified and average results are shown for all groups at 2 weeks postlesion. Scale bar = 250 µm. **(B):** Safranin‐O/fast green staining is shown in a representative longitudinal section of the IVD and adjacent vertebrae. Scale bar = 250 µm. **(C):** Respective histological grading scores were calculated for all groups at 2 weeks postlesion from high‐magnification histological transversal sections showing NP and AF as well as the interface (dashed line). ∗, *p* ≤ .05. Scale bar = 100 µm. Abbreviations: AF, annulus fibrosus; %DHI, percentage of disc height index; IVD, intervertebral disc; NP, nucleus pulposus; rFb, rat dermal fibroblasts; rMSC, rat bone marrow mesenchymal stem cells.

Rat dermal fibroblasts (rFbs) were isolated according to an established protocol [28]. Briefly, approximately 1 cm^2^ of underarm skin was collected, and mechanically and enzymatically dissociated with Liberase TM Research Grade (F. Hoffmann‐La Roche, Basel, Switzerland, http://www.roche.com). rFbs were cultured in Dulbecco’s modified Eagle’s medium (DMEM) with 15% FBS (Lonza, Basel, Switzerland, http://www.lonza.com) and expanded up to P5. Their phenotypic profile and multilineage differentiation potential was assessed along with rMSCs (supplemental online Fig. 1). For transplantation 2 × 10^6^ cells at P5 were resuspended in 400 µl of saline solution and administered by injection into the lateral tail vein using a 24‐gauge catheter (B. Braun, Melsungen, Germany, https://www.bbraun.com), under anesthesia.

### Tissue Collection

Animals were killed by intracardiac exsanguination under anesthesia. Whole blood was collected with anticoagulant citrate‐phosphate‐dextrose‐adenine solution (Sigma‐Aldrich, St. Louis, MO, http://www.sigmaaldrich.com) and processed for flow cytometry. After dissection, the draining lymph nodes (iliac and inguinal) and the spleen were collected in DMEM and processed for flow cytometry. The NP of Co5/6 and Co7/8 were collected in RNAlater (Thermo Fisher) and kept at −80°C until RNA isolation. For histological analysis, Co6/7 was collected en bloc with adjacent vertebrae and the section was fixed, decalcified, and processed for paraffin embedding. Consecutive transversal 5‐µm slices of the IVD were collected in a microtome (Leica Biosystems, Buffalo Grove, IL, http://www.leicabiosystems.com).

### Calculation of Disc Height Index

Digital radiographs were acquired by the Owandy‐RX radiology system equipped with the Opteo digital sensor (Owandy Radiology, Oxford, CT, http://www.owandy.com) and processed with the QuickVision software. The percentage of the disc height index (%DHI) was calculated using the ImageJ software (https://imagej.nih.gov/ij), by the disc height index ratio between postpuncture and prepuncture [%DHI = (postpunctured DHI/prepunctured DHI) × 100], as previously described [29].

### Histological Analysis

For histological grading score, tissue slices were stained with Safranin‐O and evaluated for five categories of IVD degenerative alterations: AF cellularity, AF morphology, border between AF and NP, NP cellularity, and NP morphology; each was scored between 1 (normal disc) and 5 (highly degenerated disc). The sum of each score per category was calculated. For determination of hernia volume and ECM content, Safranin‐O and Masson’s trichrome stainings were performed throughout the IVD length. Hernia volume was measured from the Safranin‐O stained sections as the sum of areas of each individual section throughout the IVD, as previously described [27]. For determination of the percentage of area (%area) of proteoglycans and collagen, a custom ImageJ macro, based on a color deconvolution technique, was used to separate color channels from Safranin‐O and Masson's trichrome stainings, respectively [30].

### Immunofluorescence and Immunohistochemistry

For collagen type II, tissue slices were incubated with the primary antibody anti‐collagen II‐II6B3 (Developmental Studies Hybridoma Bank, Iowa City, IA, http://dshb.biology.uiowa.edu), followed by incubation with the secondary antibody Alexa Fluor 488 goat anti‐mouse (Thermo Fisher). The total number of positive pixels per tissue area was determined as a percentage of fluorescence divided by the total pixels of each image tile, using Interactive Data Language version 8.2 (IDL; Exelis Visual Information Solutions, Inc./Harris Corp., http://www.exelisvis.com). For the following antibodies, the Novolink Max‐Polymer detection system (Leica Biosystems) was used, according to the manufacturer’s instructions: anti‐aggrecan (Santa Cruz Biotechnology, Santa Cruz, CA, https://www3.scbt.com), anti‐fibronectin (Santa Cruz), anti‐Ki‐67 (Thermo Scientific Life Sciences), anti‐MMP3 (Abcam, Cambridge, MA, http://www.abcam.com), anti‐MMP14 (Abcam), anti‐CD68 clone ED1 (Bio‐Rad Laboratories, Hercules, CA, https://www.bio‐rad‐antibodies.com), and anti‐Pax5 (Leica Biosystems).

### RNA Isolation and Reverse Transcription Quantitative Real‐Time Polymerase Chain Reaction

Total RNA was isolated from the NP of Co5/6 and Co7/8 using TRIzol reagent. RNA was quantified by Nanodrop (Thermo Fisher), RNA integrity was assessed by denaturing agarose gel electrophoresis, and samples were treated with DNase (Turbo DNA‐free Kit; Thermo Fisher). Complementary DNA (cDNA) was obtained through the high‐capacity cDNA reverse transcription kit, according to the manufacturer's instructions (Applied Biosystems, Foster City, CA, http://www.appliedbiosystems.com). Quantitative polymerase chain reaction (qPCR) was carried out in an iQ5 Real‐Time PCR Detection System (Bio‐Rad Laboratories) using TaqMan Gene Expression Master Mix and TaqMan Gene Expression Assays (Applied Biosystems), namely: collagen type I (COL1A1): Rn01463848_m1; COL2A1: Rn01637087_m1; matrix metalloproteinase 3 (MMP3): Rn00591740_m1; cluster of differentiation 24 (CD24): Rn00562598_m1; keratin 19 (KRT19): Rn01496867_m1, Slc2a1 (glucose transporter 1 [GLUT‐1]): Rn01417099_m1; and GAPDH: Rn99999916_s1, as a reference gene. Experiments were performed in duplicate and a quantification cycle (Cq) 35 cutoff was used. Relative expression levels were calculated using the Cq method, according to Minimum Information for Publication of Quantitative Real‐Time PCR Experiments guidelines [31].

### Flow Cytometry Analysis

Whole blood was overlaid on Lymphoprep (Axis‐Shield, Dundee, U.K., http://www.axis‐shield.com) in a 1:1 ratio. Samples were centrifuged and plasma stored at −80°C for cytokine analysis. Peripheral blood mononuclear cells were collected from the interface. Lymph nodes were mechanically dissociated and spleen was enzymatically and mechanically dissociated. Red blood cells were lysed, blocked, and incubated with the following anti‐rat antibodies: CD45R‐PE (clone HIS24), TCR‐PerCP (clone R73), major histocompatibility complex class II (MHCII)‐Alexa Fluor 647 (clone OX‐6), CD4‐APC (clone OX35), and CD161a‐FITC (clone 10/78) (BD, Franklin Lakes, NJ, http://www.bd.com). Samples were acquired on a flow cytometer (FACSCanto II; BD) and data analyzed with the FlowJo software version 8.7 (FlowJo, Ashland, OR, http://www.flowjo.com). Representative plots of the stainings are in supplemental online Fig. 2.

**Figure 2:**
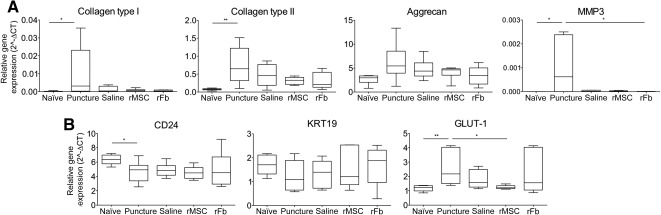
Real‐time quantitative polymerase chain reaction gene expression analysis within the nucleus pulposus for the extracellular matrix components collagen type I, collagen type II, aggrecan, and MMP3 **(A)** and for the nucleus pulposus cell markers CD24, KRT19, and GLUT‐1 **(B)**. Results were obtained from the mean of technical duplicate measurements and are presented as relative gene expression to the endogenous reference gene GAPDH. Box‐and‐whisker plots showing minimum to maximum values are represented. ∗, *p* ≤ .05; ∗∗, *p* ≤ .01. Abbreviations: 2^‐ΔCT, relative gene expression; MMP3, matrix metalloproteinase 3; rFb, rat dermal fibroblasts; rMSC, rat bone marrow mesenchymal stem cells.

### Plasma Cytokine Quantification

The membrane‐based Rat Cytokine Antibody Array C2 (RayBiotech, Norcross, GA, http://www.raybiotech.com) was used for semiquantitative detection of cytokines in the plasma, according to the manufacturer’s instructions. We analyzed 1 ml of the plasma pool (*n* = 6) for each of the naïve, saline, rMSC, and rFb groups. Signal density for each sample spot was determined using Chemidoc XRS+ (Bio‐Rad Laboratories) and ImageJ software. Relative cytokine levels were normalized to the positive control and to the reference näive group. FasL, fractalkine, and B7‐2/CD86 were not detected in the näive group, so for these, an arbitrary value of 1.5 instead of 0 was considered for calculation of fold‐change.

### Statistical Analysis

Results were expressed as mean ± SD and statistical analysis was performed using GraphPad Prism software (San Diego, CA, http://www.graphpad.com), with statistical significance set at *p* ≤ .05. Normality was assessed by the D'Agostino‐Pearson omnibus normality test. For all data sets, analysis of differences between samples was made by the nonparametric Kruskal‐Wallis test, followed by Dunn's multiple comparison test, except for the %DHI analysis, in which one‐way analysis of variance followed by Tukey's post hoc test was used.

## Results

A rat caudal IVD lesion model developed previously in our laboratory [27] was used to analyze the potential of systemically transplanted MSCs to reduce disc degeneration. Lesion was induced by a 21‐gauge needle puncture to the coccygeal discs 5/6, 6/7, and 7/8, and 2 weeks was allowed for degeneration to progress. At 24 hours after the lesion was made, animals had intravenous transplantation of either rMSCs or rFbs isolated from littermate rats. Control groups consisted of puncture without transplantation or administration of vehicle solution (saline). A näive unlesioned group was analyzed in parallel.

### Evaluation of Degeneration Within the IVD by Radiological, Histological, and Gene Expression Analyses

Radiographs were collected for each disc immediately before and 2 weeks after puncture. %DHI was calculated as a value inversely proportional to the degree of disc degeneration (Fig. 1A). Results showed that the rMSC group’s %DHI was closer to that of the naïve group, although the difference was not statistically significant.

For quantification of the degree of degeneration within the disc, we used a histological grading scale that evaluates AF cellularity and morphology, the border between AF and NP, and NP cellularity and morphology, with each feature scored between 1 (normal disc) and 5 (highly degenerated disc) [29]. The additive score per group is presented in Figure 1B. The most obvious alterations were found for the border between AF and NP (Fig. 1C; supplemental online Table 1). Results showed a statistically significant degeneration for the puncture alone and puncture+rFb groups relative to the naïve group. This difference was not evident for the saline and rMSC groups. The smallest mean value of degeneration, found for the rMSC group, together with the highest value of %DHI, suggested a smaller degree of disc degeneration when MSCs were transplanted.

Gene expression within the IVD was analyzed for major ECM components, including collagen type I (Col1a1), collagen type II (Col2a1), aggrecan, and the matrix metalloproteinase 3 (MMP3), which has been reported as a major factor responsible for disc ECM breakdown and related to IVD degeneration [32]. As shown in Figure 2A, at 2 weeks after injury, the major effect was found for group puncture, with statistically significant upregulation of Col1a1, Col2a1, and MMP3. The upregulation of the expression of all major ECM components may reflect cells’ attempt to balance these proteins in response to ongoing degenerative changes at this time point [33]. Importantly, these differences disappeared for the treated groups, indicating that the treatments lowered the expression levels of these genes to physiological levels (i.e., similar to naïve), which may indicate less severe degeneration in these groups.

Moreover, we also analyzed CD24, KRT19, and GLUT‐1—rat NP markers [34, 35]. As shown in Figure 2B, all groups showed a downregulation in CD24, which was due to the lesion induced, whereas no difference between groups was observed for KRT19. The lesion also induced an upregulation of GLUT‐1, which has been associated with hypoxia and rat IVD degeneration [34, 35]. Importantly, this upregulation was reverted in the rMSC group, indicating a beneficial effect on NP cells by the transplanted cells.

**Figure 3:**
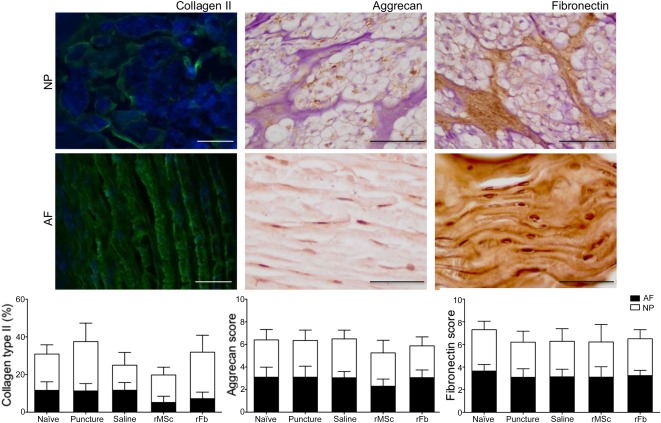
Analysis of extracellular matrix components collagen type II, aggrecan, and fibronectin within the NP and AF. Collagen type II immunofluorescence is shown in green; 4′,6‐diamidino‐2‐phenylindole nuclear staining is shown in blue. Quantification was performed as a percentage of positive pixels per area of tissue. Scale bar = 100 µm. Aggrecan and fibronectin immunohistochemistry is shown in brown, with hematoxylin counterstaining in purple. Staining semiquantification was done by attributing a score, as follows: 1 = extremely weak, 2 = weak, 3 = moderate, 4 = strong, 5 = extremely strong. Scale bar = 50 µm. Abbreviations: AF, annulus fibrosus; NP, nucleus pulposus.

Finally, collagen II, aggrecan, and fibronectin, main ECM components, were quantified within the IVD at the protein level. Aggrecan and fibronectin displayed mainly a pericellular localization but were also found in the cytoplasm, whereas collagen II could be detected mainly extracellularly (Fig. 3). No statistical differences between groups were found, although the rMSC group showed a tendency for a smaller amount of collagen II in both AF and NP. These results also showed that the increase in collagen II and aggrecan observed at the gene level did not translate into an increased protein production at 2 weeks.

### Hernia Histopathological Evaluation Through Analysis of ECM Composition and Inflammatory Infiltrates

Ongoing degeneration can lead to herniation, stenosis, segmental instability, and degenerative scoliosis. In this model, we have previously reported the formation of hernia in response to injury [27].

**Figure 4:**
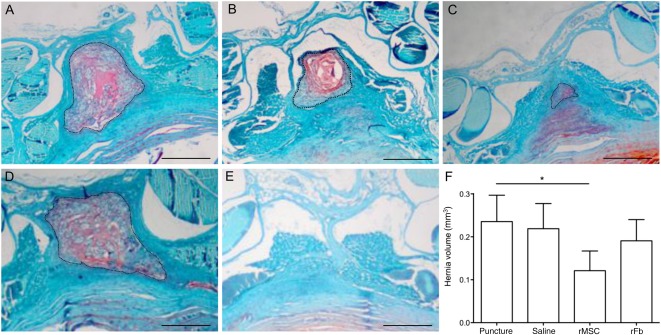
Intervertebral disc (IVD) herniation. Representative sections of the hernia for the following groups: puncture **(A)**, saline **(B)**, rMSC **(C)**, and rFb **(D)**, showing proteoglycan‐rich extracellular matrix (red), corresponding to the herniated tissue, and respective control tissue for the naïve group **(E)**. **(F):** Hernia volume (mm^3^), calculated from the sum of the areas of each individual section throughout the IVD, is shown for all groups. Dashed line outlines the hernia in the section. ∗, *p* ≤ .05. Scale bar = 500 µm. Abbreviations: rFb, rat dermal fibroblasts; rMSC, rat bone marrow mesenchymal stem cells.

The herniated tissue formed 2 weeks after lesion was quantified from hernia area delimitation on tissue slices at precise intervals throughout the IVD. Hernia volume was calculated and results presented as mean hernia volume per group (Fig. 4). Noteworthy is that one of six animals in the rMSC group and one of six animals in the saline group had no hernia, and in the puncture and rFB groups, all animals had hernias. Results represent six animals per group, including the zero value for animals with no hernia. Interestingly, animals in the group transplanted with rMSCs had significantly smaller hernias compared with animals with puncture alone, whereas the saline and rFB groups had hernias with dimensions similar to that of the puncture group (Fig. 4F). Plots for the complete hernia profiles are shown in supplemental online Figure 3.

**Figure 5:**
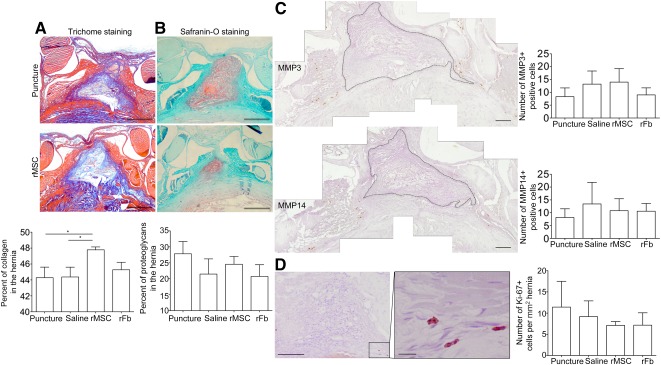
Quantification of extracellular matrix components (collagen type I and proteoglycans) within the hernia. The percentage area within the hernia occupied by collagen type I (quantified from Masson's trichrome staining) **(A)** and proteoglycans (quantified from Safranin‐O staining) **(B)**. Scale bar = 500 µm. ∗, *p* ≤ .05. **(C):** Quantification of MMP3 and MMP14 immunoreactivity within the hernia site. Positive cells for both MMPs were found at the extreme borders of the hernia, as shown here for the rMSC group. Hematoxylin counterstaining is shown in purple. Dashed line delimitates the hernia. Scale bar = 250 µm. **(D):** Cell proliferation within the herniated tissue, showing Ki‐67 expression in brown in low and high magnification and respective quantification. Hematoxylin counterstaining is shown in purple. Scale bar = 100 µm; scale bar in the inset = 10 µm. Abbreviations: MMP, matrix metalloproteinase; rFb, rat dermal fibroblasts; rMSC, rat bone marrow mesenchymal stem cells.

To understand the hernia ECM composition, %area of collagen type I and proteoglycans, main IVD extracellular components, were quantified within the herniated tissue. Results indicated a significant increase in collagen I for the rMSC group (Fig. 5A), whereas no differences were found for proteoglycans (Fig. 5B). MMP3 and MMP14 were also quantified at the hernia site and positive cells were found exclusively at the extreme borders of the hernia with no differences between groups (Fig. 5C).

For cell proliferation analysis, we found Ki‐67+ cells exclusively at the borders of the hernia with no significant differences between groups, although the treated groups had slightly reduced cell proliferation with respect to puncture alone (Fig. 5D).

**Figure 6:**
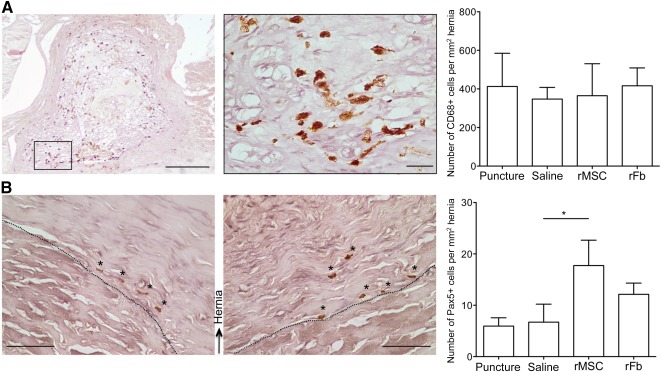
Macrophage and B‐lymphocyte immunoreactivity within the hernia. **(A):** CD68+ macrophages are shown in brown at low and high magnification, with respective quantification. Scale bar = 200 µm; scale bar in the inset = 20 µm. **(B):** Pax5+ B lymphocytes (*) are shown at the borders of the hernia for the saline group (left) and the rMSC group (right), with respective quantification. ∗, *p* ≤ .05. Hematoxylin counterstaining is shown in purple. Dashed line delimitates the hernia. Scale bar = 50 µm. Abbreviations: rFb, rat dermal fibroblasts; rMSC, rat bone marrow mesenchymal stem cells.

We and others have shown that hernia can spontaneously recede. This phenomenon has been attributed to the inflammatory response from the host tissue, in particular that associated with macrophage activity. Given that the group transplanted systemically with rMSCs showed reduced hernia volume at 2 weeks postlesion (Fig. 4), we hypothesized that the reduced hernia could be associated with the immune response. To explore this hypothesis, we analyzed the presence of macrophages and B lymphocytes within the hernia, by measuring immunoreactivity against CD68(ED1) and Pax5, respectively. For all hernias analyzed, we found CD68+ exclusively within the hernia, evenly distributed and in high abundance, with no positivity found elsewhere in the section (Fig. 6A), whereas Pax5+ cells were found almost exclusively at the borders of the hernia and in low number. Although the results showed no difference in macrophage presence among the groups, we observed a significant increase in B lymphocytes in the rMSC transplanted group. To better understand how rMSCs transplanted systemically could induce a reduction in IVD degeneration and herniation, through increase in B lymphocytes, we analyzed the systemic immune response.

### Analysis of the Systemic Immune Response Associated With MSC Transplantation

A balanced immune response following lesion is critical to the overall regenerative process. Here, the presence of the main immune cell populations, myeloid cells (including monocytes, macrophages, and dendritic cells), and B and T lymphocytes, was analyzed by flow cytometry in the blood, draining lymph nodes, and spleen. Moreover, analysis of a set of inflammatory mediators in the plasma was performed by antibody array.

**Figure 7:**
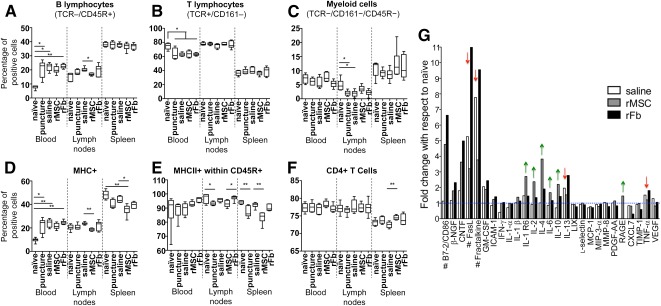
Analysis of the systemic immune response for blood, draining lymph nodes, and spleen, by flow cytometry **(A–F)**, and semiquantitative antibody detection of an array of inflammatory mediators **(G)**. The following main immune cell populations were analyzed: B lymphocytes **(A)**, T lymphocytes **(B)**, and myeloid cells **(C)**, as well as MHCII‐expressing cells **(D)**, percentage of B lymphocytes expressing MHCII **(E)**, and percentage of T lymphocytes expressing CD4, which corresponds to populations of regulatory and helper T lymphocytes **(F)**. Box‐and‐whisker plots are represented showing minimum to maximum values. ∗, *p* ≤ .05. ∗∗, *p* ≤ .01. **(G):** Semiquantitative antibody detection of an array of inflammatory mediators in the rat plasma for the saline, rMSC, and rFb groups, expressed as fold‐change with respect to the naïve group. #, FasL, fractalkine, and B7‐2/CD86 were not detected in the naïve group; for these, an arbitrary value of 1.5 was considered for calculation of fold‐change for the remaining groups. The gridline at *y* = 1 corresponds to baseline naïve values. Arrows indicate an upregulation (green) or downregulation (red) for the rMSC group with respect to the saline and rFb groups. Abbreviations: CNTF, ciliary neurotrophic factor; CXCL7, chemokine (C‐X‐C motif) ligand 7; GM‐CSF, granulocyte‐macrophage colony‐stimulating factor; ICAM, intercellular adhesion molecule; IFN, interferon; IL, interleukin; MCP, monocyte chemoattractant protein; MHCII, MHC class II; MIP, macrophage inflammatory protein; MMP, matrix metalloproteinase; NGF, nerve growth factor; PDGF, platelet‐derived growth factor; RAGE, receptor for advanced glycation end products; rFb, rat dermal fibroblasts; rMSC, rat bone marrow mesenchymal stem cells; TIMP, tissue inhibitor of metalloproteinases; TNF, tumor necrosis factor; VEGF, vascular endothelial growth factor.

The results indicated that the puncture per se resulted in an increase in B cells (Fig. 7A) and decrease in T cells (Fig. 7B) in the blood, as well as in a decrease in myeloid cells in the lymph nodes (Fig. 7C). Interestingly, the rMSC group showed percentages of blood B lymphocytes closer to that of the naïve group, losing the statistical difference that existed between the naïve group and the remaining groups. Moreover, the percentage of B cells in the lymph nodes of the rMSC group was significantly lower compared with the saline group, which also places the rMSC group closer to the naïve group (Fig. 7A).

We also assessed the presence of professional antigen‐presenting cells, including myeloid cells and B lymphocytes, by quantifying the percentage of cells expressing MHCII. We observed a significant increase in MHCII+ cells in the blood for all groups except the rMSC group versus the naïve group (Fig. 7D). This was accompanied by a reduction of these cells in the spleen of all lesioned animals; this was only significant, however, for the rMSC group when compared with the naïve or saline groups. For the rMSC group, we also found a significant decrease in MHCII+ cells in the lymph nodes and spleen, compared with that in the saline group (Fig. 7D). When analyzing MHCII+ B cells in the blood, only the rFb group showed a significant increase in relation to the puncture or saline groups; lymph nodes show a similar pattern to spleen, with significant reductions in the puncture group relative to the naïve group, and in the rMSC group compared with the rFb group (lymph nodes) and saline group (spleen) (Fig. 7E).

Within the populations of T lymphocytes, the percentage of CD4+ cells, which includes T helper (Th) and regulatory T (Treg) cells, was quantified. Animals with rMSCs had significantly more CD4+ T cells in the spleen than the saline group (Fig. 7F). Finally, we quantified natural killer (NK)+ and natural killer T (NKT)+ cells by staining against CD161 and TCR; no differences were observed for blood or spleen, whereas in the lymph nodes, levels were too low to be conclusive (data not shown).

These results indicated that MSC systemic transplantation decreased general activation of antigen‐presenting cells, which is in line with the immunomodulatory effects of MSCs. Moreover, the increase in CD4+ cells when MSCs are transplanted is compatible with an increase in Treg cells, although our analysis was not able to discriminate the percentage of Treg cells from Th cells.

To better understand the systemic response after puncture and respective treatments, we analyzed the secretion of a set of inflammatory mediators in the plasma, using a semiquantitative antibody array. The factors analyzed included chemokines, growth factors, cell adhesion molecules, and pro‐ and anti‐inflammatory cytokines (Fig. 7G). The most significant effect was found for B7‐2/CD86, FasL, and fractalkine, which were not detected in the naïve group but were present in all three lesioned groups. B7‐2/CD86 was upregulated specifically in the cell‐transplanted groups. On the other hand, FasL and fractalkine were both downregulated in the rMSC group compared with the saline and rFb groups. Also, in the rMSC group, the proinflammatory cytokines interleukin (IL)‐13 and tumor necrosis factor (TNF)‐α were downregulated. Additionally, we found an upregulation of the cytokines IL‐2, IL‐4, IL‐6, and IL‐10, and the cytokine receptor IL‐1 R6 in the rMSC group. Finally, the rMSC group was the only group in which the soluble form of receptor for advanced glycation end products (RAGE) was detected.

Overall, MSCs seemed to be downregulating proinflammatory factors and upregulating anti‐inflammatory factors, once again confirming their immunomodulatory role in our IVD lesion model. Although this systemic effect does not directly explain the reduction in IVD degeneration and herniation in situ, it may help identify clues to the relationship between local and systemic response in tissue regeneration.

## Discussion

This study focuses on systemic transplantation of allogeneic bone marrow MSCs, exploring IVD tissue regeneration via endogenous cell migration and/or signaling to the lesion site. MSC transplantation was performed 24 hours after inducing a lesion into a rat IVD caudal lesion model previously developed by our team [27], because we intended to target the acute phase of degeneration and because it has been suggested that MSCs beneficial effect is greater at this time point [36, 37].

Recent studies indicate that MSCs can be recruited in response to IVD lesions [23, 24, 25]; paracrine communication with the IVD has been less explored [26]. Our results showed that MSC systemic transplantation results in inhibition of IVD degeneration and herniation, and demonstrated that MSCs are able to communicate with the IVD inflammatory microenvironment and modulate it. Of note, our results clearly showed a difference between the effects of rMSCs and rFbs in the IVD, demonstrating MSCs specifically have a beneficial role in IVD pathophysiology.

In the scope of this study, we have not traced the fate of transplanted MSCs, because their labeling (via luciferase transfection or with fluorescent dyes) could compromise their viability and physiological functions in vivo, and markers for tracking unlabeled MSCs in vivo are lacking [38]. Nevertheless, mechanistically, most of the positive effects reported for MSC therapies have been attributed to their paracrine effects rather than their faculty to migrate and transdifferentiate in the lesion site [39]. This could be because MSCs have a relatively short life after intravenous transplantation in vivo and engraftment rates are known to be low due to the large number of cells that become trapped in the lungs [40, 41].

The phenomenon of hernia regression has been associated with the inflammatory response from the host tissue [42, 43]. In particular, macrophages derived from monocytes, which migrate to the hernia, are key players in hernia regression, due to their capability to actively phagocytize the herniated tissue and process it in their lysosomes filled with collagen‐degrading enzymes [43]. Macrophages also secrete lysosomal enzymes by exocytosis, thus breaking down intercellular substances [44]. Surprisingly, we found an increase in Pax5+ B lymphocytes in situ in the hernia, but CD68+ macrophages were not altered between groups. The constant percentage of CD68+ cells in the hernia may occur because only a certain number of cells will be recruited and activated per area of hernia, maintaining the tissue homeostasis. MSCs can inhibit B‐lymphocyte proliferation and differentiation [45, 46], but MSC actions are very much dependent on the specific disease‐related tissue microenvironment. Specifically, in the IVD, the presence of T and B lymphocytes in isolated human herniated discs and in experimental porcine models has been previously reported [47]. Also, in human herniated tissue, lymphocytes were found to be three times more abundant in sequestrated hernias than in extrusions, whereas no other inflammatory cells were seen in protrusions apart from macrophages [48].

A local and systemic balanced immune response after occurrence of a lesion is critical to the overall regenerative process and the equilibrium between different cells drives the tissue response to injury. In this study, the presence of the main immune cell populations, myeloid cells, and B and T lymphocytes was analyzed in the blood, lymph nodes, and spleen. The lesion itself led to alterations in the B‐ and T‐cell homeostasis in the blood, with more B and fewer T cells being present 2 weeks after inducing the lesion. This was accompanied by an increase in the percentage of MHC‐II+ cells, which could reflect the increase in B‐cell number. However, MHCII+ within the CD45R+ cells does not follow the same tendency. Interestingly, for several populations analyzed, the group transplanted with rMSCs showed a tendency toward results similar to that of the naïve group, sometimes with significant differences from the saline group, as is the case for B cells in the lymph nodes and losing the statistical significance observed in other groups for B cells in the blood. Analysis of soluble mediators in the plasma also revealed the appearance of CD86, FasL, and fractalkine. Soluble CD86 may be produced by monocytes and, in this form, it has been associated with T‐lymphocyte activation [49]. Interestingly, soluble FasL may trigger lysis of activated lymphocytes by binding to the Fas receptor and thus possibly be involved in regulation of the number of T lymphocytes. Fractalkine (also known as CX3CL1), which possesses chemotactic activity toward different immune cell populations, has been identified in the plasma for different autoimmune disorders and thus may indicate that the lesion led to an inflammatory response that was not resolved even 2 weeks later. This inflammatory response will be important for MSCs to act as immunomodulators, because their activity depends on the microenvironment that the cells encounter [12]. When transplanting MSCs systemically, we observed an increase in CD4+ T cells in the spleen. The population analyzed includes both CD4 T helper and Treg cells. Interestingly, MSCs have numerous effects on T‐cell effector pathways [50] and one of the main effects is to promote the generation of Treg cells [51, 52], in some instances leading to a four‐ to fivefold increase in the percentage of cells present in circulation [53]. IL‐2 levels were increased in the rMSC group and IL‐2 is known to stimulate both Treg and effector T cells, depending on the dose [54]. Also, transplantation of rMSCs led to a reduction of MHCII+ cells, both in general and when B cells were analyzed, and in the different organs. This could be explained by a reduction in activation of antigen‐presenting cells, because the percentage of each cell population does not vary in the same way. Apart from MSCs’ direct immunosuppressive action on T cells, they have also a modulatory effect on antigen‐presenting cells such as dendritic cells (DCs) and B cells that, in turn, results in altered cytokine expression and impaired antigen presentation. It has been shown that MSCs are, indeed, able to retain DCs in an immature state or even to induce DCs to acquire a tolerogenic phenotype, with a lower expression of MHCII molecules, among others [55]. Induction of tolerogenic DC may be induced by the IL‐6 produced by MSCs [56], which here was increased in the plasma of the rMSC group. Tolerogenic DC may contribute to production of Treg to trigger a Th2 response, leading to the observed increased levels of IL‐4 and IL‐10 and the decrease in TNF‐α level. The levels of IFN‐γ were close to that of the naïve group, further corroborating a Th2 type of response. A Th2 response could then be the reason for the increase in the percentage of B cells observed in the hernia of rMSC animals. IL‐13 was reduced in the rMSC group, correlating with the low expression of MHCII on B cells [57]. This inflammatory cytokine is a mediator of tissue fibrosis in inflammatory contexts such as schistosomiasis and asthma [57]. Thus, its reduction, as well as the decrease in FasL and fractalkine, further points toward the immunomodulatory role of MSCs. Interestingly, soluble RAGE was found only in the rMSC group, in agreement with what has been reported 1 and 4 weeks after systemic administration of MSCs to treat ischemic stroke rats [58]. RAGE is a receptor that binds multiple ligands that accumulate in a damaged cell environment; thus it has been hypothesized that soluble RAGE will sequester RAGE ligands, thus blocking their interaction with RAGE and diminishing inflammation.

Comparing rMSC and rFb transplantation, there was a clear difference between the animals’ immunologic responses. Although dermal fibroblasts have been suggested to display similar phenotypic and differentiation capacities as MSCs, previous reports have clearly shown that these cell types differ considerably in their anti‐inflammatory potential [59]. In our hands, however, they showed a marked difference in terms of differentiation potential.

Although the immunomodulatory role of MSCs has long been suggested, further studies will need to address the mechanism by which MSCs have a positive effect on IVD regeneration, and the role of B lymphocytes in MSC‐mediated hernia reduction. Moreover, IVD cells are a heterogeneous cell population, containing fibroblast‐like cells and chondrocyte‐like cells but also progenitor cells, that sustains a possible endogenous regenerative capacity of the IVD [60, 61]. This also illustrates the complexity of the IVD tissue, which must be considered when envisaging clinical translation of the results obtained from animal models, as well as the different mechanical loading and sizes of the discs, the presence of notochordal cells within the NP, the unequal growth of the epiphyseal plate, and differences in the inflammatory environment of the disc.

## Conclusion

This study highlights the communication between the proinflammatory IVD microenvironment and the immunomodulatory ability of MSCs in regulating the local and systemic inflammatory response during IVD degeneration. It also highlights the complexity of interactions that occur between stromal and immune cells and their interplay toward achieving a balanced IVD regeneration.

## Author Contributions

C.C.: conception and design, provision of study material, collection and assembly of data, data analysis and interpretation, manuscript writing, final approval of manuscript; C.R.A.: provision of study material, collection and assembly of data, data analysis and interpretation, manuscript writing, final approval of manuscript; M.I.A.: provision of study material, collection and assembly of data, data analysis and interpretation, final approval of manuscript; A.M.S.: collection of data, data analysis and interpretation, final approval of manuscript; M.M., S.L., C.L.P., and G.Q.T.: collection of data, final approval of manuscript; A.T.M.: assembly of data, data analysis, final approval of manuscript; S.G.S.: conception and design, data analysis and interpretation, final approval of manuscript; R.M.G.: conception and design, data analysis and interpretation, manuscript writing, final approval of manuscript; M.A.B.: conception and design, financial support, final approval of manuscript.

## Disclosure of Potential Conflicts of Interest

The authors indicated no potential conflicts of interest.

## Supporting information

Supporting Information

## References

[1] Molinos M , Almeida CR , Caldeira J et al. Inflammation in intervertebral disc degeneration and regeneration. J R Soc Interface 2015;12:20141191. 25673296 10.1098/rsif.2014.1191 PMC4345483

[2] Sivan SS , Hayes AJ , Wachtel E et al. Biochemical composition and turnover of the extracellular matrix of the normal and degenerate intervertebral disc. Eur Spine J 2014;23(suppl 3)S344–353. 23591805 10.1007/s00586-013-2767-8

[3] Silva‐Correia J , Correia SI , Oliveira JM et al. Tissue engineering strategies applied in the regeneration of the human intervertebral disk. Biotechnol Adv 2013;31:1514–1531. 23911974 10.1016/j.biotechadv.2013.07.010

[4] Sakai D , Andersson GB . Stem cell therapy for intervertebral disc regeneration: Obstacles and solutions. Nat Rev Rheumatol 2015;11:243–256. 25708497 10.1038/nrrheum.2015.13

[5] Ma S , Xie N , Li W et al. Immunobiology of mesenchymal stem cells. Cell Death Differ 2014;21:216–225. 24185619 10.1038/cdd.2013.158 PMC3890955

[6] Clarke LE , Richardson SM , Hoyland JA . Harnessing the potential of mesenchymal stem cells for IVD regeneration. Curr Stem Cell Res Ther 2015;10:296–306. 25440320 10.2174/1574888x10666141202112638

[7] Le Visage C , Kim SW , Tateno K et al. Interaction of human mesenchymal stem cells with disc cells: Changes in extracellular matrix biosynthesis. Spine 2006;31:2036–2042. 16915085 10.1097/01.brs.0000231442.05245.87

[8] Svanvik T , Henriksson HB , Karlsson C et al. Human disk cells from degenerated disks and mesenchymal stem cells in co‐culture result in increased matrix production. Cells Tissues Organs 2010;191:2–11. 19494482 10.1159/000223236

[9] Hiyama A , Mochida J , Iwashina T et al. Transplantation of mesenchymal stem cells in a canine disc degeneration model. J Orthop Res 2008;26:589–600. 18203202 10.1002/jor.20584

[10] Marfia G , Campanella R , Navone SE et al. Potential use of human adipose mesenchymal stromal cells for intervertebral disc regeneration: A preliminary study on biglycan‐deficient murine model of chronic disc degeneration. Arthritis Res Ther 2014;16:457. 25293819 10.1186/s13075-014-0457-5 PMC4223513

[11] Omlor GW , Fischer J , Kleinschmitt K et al. Short‐term follow‐up of disc cell therapy in a porcine nucleotomy model with an albumin‐hyaluronan hydrogel: In vivo and in vitro results of metabolic disc cell activity and implant distribution. Eur Spine J 2014;23:1837–1847. 24801573 10.1007/s00586-014-3314-y

[12] Wang Y , Chen X , Cao W et al. Plasticity of mesenchymal stem cells in immunomodulation: Pathological and therapeutic implications. Nat Immunol 2014;15:1009–1016. 25329189 10.1038/ni.3002

[13] Clinical US National Institutes of Health. Safety and preliminary efficacy study of mesenchymal precursor cells (MPCs) in subjects with lumbar back pain. Available at https://clinicaltrials.gov/ct2/show/NCT01290367. Accessed January 18, 2016..

[14] Huang YC , Leung VY , Lu WW et al. The effects of microenvironment in mesenchymal stem cell‐based regeneration of intervertebral disc. Spine J 2013;13:352–362. 23340343 10.1016/j.spinee.2012.12.005

[15] Teixeira GQ et al. A degenerative/proinflammatory intervertebral disc organ culture: An ex vivo model for anti‐inflammatory drug and cell therapy. Tissue Eng Part C Methods 2016;22:8–19. 26565141 10.1089/ten.tec.2015.0195

[16] Vadalà G , Sowa G , Hubert M et al. Mesenchymal stem cells injection in degenerated intervertebral disc: Cell leakage may induce osteophyte formation. J Tissue Eng Regen Med 2012;6:348–355. 21671407 10.1002/term.433

[17] Sakai D , Grad S . Advancing the cellular and molecular therapy for intervertebral disc disease. Adv Drug Deliv Rev 2015;84:159–171. 24993611 10.1016/j.addr.2014.06.009

[18] Grad S , Peroglio M , Li Z et al. Endogenous cell homing for intervertebral disk regeneration. J Am Acad Orthop Surg 2015;23:264–266. 25808688 10.5435/JAAOS-D-15-00096

[19] Li Z , Peroglio M , Alini M et al. Potential and limitations of intervertebral disc endogenous repair. Curr Stem Cell Res Ther 2015;10:329–338. 25741710 10.2174/1574888x10666150305105114

[20] Fong EL , Chan CK , Goodman SB . Stem cell homing in musculoskeletal injury. Biomaterials 2011;32:395–409. 20933277 10.1016/j.biomaterials.2010.08.101 PMC2991369

[21] Almeida CR , Vasconcelos DP , Goncalves RM et al. Enhanced mesenchymal stromal cell recruitment via natural killer cells by incorporation of inflammatory signals in biomaterials. J R Soc Interface 2012;9:261–271. 21752807 10.1098/rsif.2011.0357 PMC3243397

[22] Anton K , Banerjee D , Glod J . Macrophage‐associated mesenchymal stem cells assume an activated, migratory, pro‐inflammatory phenotype with increased IL‐6 and CXCL10 secretion. PLoS One 2012;7:e35036. 22496888 10.1371/journal.pone.0035036 PMC3319627

[23] Illien‐Jünger S , Pattappa G , Peroglio M et al. Homing of mesenchymal stem cells in induced degenerative intervertebral discs in a whole organ culture system. Spine 2012;37:1865–1873. 22433498 10.1097/BRS.0b013e3182544a8a

[24] Pereira CL , Gonçalves RM , Peroglio M et al. The effect of hyaluronan‐based delivery of stromal cell‐derived factor‐1 on the recruitment of MSCs in degenerating intervertebral discs. Biomaterials 2014;35:8144–8153. 24969636 10.1016/j.biomaterials.2014.06.017

[25] Sakai D , Nishimura K , Tanaka M et al. Migration of bone marrow‐derived cells for endogenous repair in a new tail‐looping disc degeneration model in the mouse: A pilot study. Spine J 2015;15:1356–1365. 25459743 10.1016/j.spinee.2013.07.491

[26] Strassburg S , Hodson NW , Hill PI et al. Bi‐directional exchange of membrane components occurs during co‐culture of mesenchymal stem cells and nucleus pulposus cells. PLoS One 2012;7:e33739. 22438989 10.1371/journal.pone.0033739 PMC3305345

[27] Cunha C , Lamas S , Goncalves RM et al. Joint analysis of IVD herniation and degeneration by rat caudal needle puncture model. J Orthop Res 2015 [Epub ahead of print]. 10.1002/jor.23114 26610284

[28] Seluanov A , Vaidya A , Gorbunova V . Establishing primary adult fibroblast cultures from rodents. J Vis Exp 2010;:44 e2033. 10.3791/2033 PMC3185624 20972406

[29] Han B , Zhu K , Li FC et al. A simple disc degeneration model induced by percutaneous needle puncture in the rat tail. Spine 2008;33:1925–1934. 18708924 10.1097/BRS.0b013e31817c64a9

[30] Ruifrok AC , Johnston DA . Quantification of histochemical staining by color deconvolution. Ana Quant Cytol Histol 2001;23:291–299. 11531144

[31] Bustin SA , Benes V , Garson JA et al. The MIQE guidelines: Minimum information for publication of quantitative real‐time PCR experiments. Clin Chem 2009;55:611–622. 19246619 10.1373/clinchem.2008.112797

[32] Vo NV , Hartman RA , Yurube T et al. Expression and regulation of metalloproteinases and their inhibitors in intervertebral disc aging and degeneration. Spine J 2013;13:331–341. 23369495 10.1016/j.spinee.2012.02.027 PMC3637842

[33] Zhang H , Wang L , Park JB et al. Intradiscal injection of simvastatin retards progression of intervertebral disc degeneration induced by stab injury. Arthritis Res Ther 2009;11:R172. 19912653 10.1186/ar2861 PMC3003500

[34] Rajpurohit R , Risbud MV , Ducheyne P et al. Phenotypic characteristics of the nucleus pulposus: Expression of hypoxia inducing factor‐1, glucose transporter‐1 and MMP‐2. Cell Tissue Res 2002;308:401–407. 12107433 10.1007/s00441-002-0563-6

[35] Rodrigues‐Pinto R , Richardson SM , Hoyland JA . Identification of novel nucleus pulposus markers: Interspecies variations and implications for cell‐based therapies for intervertebral disc degeneration. Bone Joint Res 2013;2:169–178. 23958792 10.1302/2046-3758.28.2000184 PMC3747513

[36] Chen J , Li Y , Wang L et al. Therapeutic benefit of intravenous administration of bone marrow stromal cells after cerebral ischemia in rats. Stroke 2001;32:1005–1011. 11283404 10.1161/01.str.32.4.1005

[37] Schenk S , Mal N , Finan A et al. Monocyte chemotactic protein‐3 is a myocardial mesenchymal stem cell homing factor. Stem Cells 2007;25:245–251. 17053210 10.1634/stemcells.2006-0293

[38] Bianco P , Cao X , Frenette PS et al. The meaning, the sense and the significance: Translating the science of mesenchymal stem cells into medicine. Nat Med 2013;19:35–42. 23296015 10.1038/nm.3028 PMC3998103

[39] Caplan AI . Why are MSCs therapeutic? New data: New insight. J Pathol 2009;217:318–324. 19023885 10.1002/path.2469 PMC8793150

[40] Ren G , Chen X , Dong F et al. Concise review: Mesenchymal stem cells and translational medicine: Emerging issues. Stem Cells Translational Medicine 2012;1:51–58. 23197640 10.5966/sctm.2011-0019 PMC3727691

[41] von Bahr L , Batsis I , Moll G et al. Analysis of tissues following mesenchymal stromal cell therapy in humans indicates limited long‐term engraftment and no ectopic tissue formation. Stem Cells 2012;30:1575–1578. 22553154 10.1002/stem.1118

[42] Autio RA , Karppinen J , Niinimäki J et al. Determinants of spontaneous resorption of intervertebral disc herniations. Spine 2006;31:1247–1252. 16688039 10.1097/01.brs.0000217681.83524.4a

[43] Kobayashi S , Meir A , Kokubo Y et al. Ultrastructural analysis on lumbar disc herniation using surgical specimens: Role of neovascularization and macrophages in hernias. Spine 2009;34:655–662. 19333096 10.1097/BRS.0b013e31819c9d5b

[44] Henson PM . Mechanisms of exocytosis in phagocytic inflammatory cells. Parke‐Davis Award Lecture. Am J Pathol 1980;101:494–511. 7004205 PMC1903647

[45] Asari S , Itakura S , Ferreri K et al. Mesenchymal stem cells suppress B‐cell terminal differentiation. Exp Hematol 2009;37:604–615. 19375651 10.1016/j.exphem.2009.01.005 PMC2747661

[46] Corcione A , Benvenuto F , Ferretti E et al. Human mesenchymal stem cells modulate B‐cell functions. Blood 2006;107:367–372. 16141348 10.1182/blood-2005-07-2657

[47] Geiss A , Larsson K , Rydevik B et al. Autoimmune properties of nucleus pulposus: an experimental study in pigs. Spine 2007;32:168–173. 17224810 10.1097/01.brs.0000251651.61844.2d

[48] Virri J , Grönblad M , Seitsalo S et al. Comparison of the prevalence of inflammatory cells in subtypes of disc herniations and associations with straight leg raising. Spine 2001;26:2311–2315. 11679814 10.1097/00007632-200111010-00004

[49] Shi HZ , Xie ZF , Deng JM et al. Soluble CD86 protein in serum samples of patients with asthma. Thorax 2004;59:870–875. 15454653 10.1136/thx.2004.021840 PMC1746836

[50] Duffy MM , Ritter T , Ceredig R et al. Mesenchymal stem cell effects on T‐cell effector pathways. Stem Cell Res Ther 2011;2:34. 21861858 10.1186/scrt75 PMC3219065

[51] Luz‐Crawford P , Kurte M , Bravo‐Alegría J et al. Mesenchymal stem cells generate a CD4+CD25+Foxp3+ regulatory T cell population during the differentiation process of Th1 and Th17 cells. Stem Cell Res Ther 2013;4:65. 23734780 10.1186/scrt216 PMC3706898

[52] Burr SP , Dazzi F , Garden OA . Mesenchymal stromal cells and regulatory T cells: The Yin and Yang of peripheral tolerance?. Immunol Cell Biol 2013;91:12–18. 23146942 10.1038/icb.2012.60

[53] Carrion F , Nova E , Ruiz C et al. Autologous mesenchymal stem cell treatment increased T regulatory cells with no effect on disease activity in two systemic lupus erythematosus patients. Lupus 2010;19:317–322. 19919974 10.1177/0961203309348983

[54] Arenas‐Ramirez N , Woytschak J , Boyman O . Interleukin‐2: Biology, design and application. Trends Immunol 2015;36:763–777. 26572555 10.1016/j.it.2015.10.003

[55] Jiang XX , Zhang Y , Liu B et al. Human mesenchymal stem cells inhibit differentiation and function of monocyte‐derived dendritic cells. Blood 2005;105:4120–4126. 15692068 10.1182/blood-2004-02-0586

[56] Deng Y , Yi S , Wang G et al. Umbilical cord‐derived mesenchymal stem cells instruct dendritic cells to acquire tolerogenic phenotypes through the IL‐6‐mediated upregulation of SOCS1. Stem Cells Dev 2014;23:2080–2092. 24730420 10.1089/scd.2013.0559

[57] Wynn TA . IL‐13 effector functions. Annu Rev Immunol 2003;21:425–456. 12615888 10.1146/annurev.immunol.21.120601.141142

[58] Yang M , Wei X , Li J et al. Changes in host blood factors and brain glia accompanying the functional recovery after systemic administration of bone marrow stem cells in ischemic stroke rats. Cell Transplant 2010;19:1073–1084. 20412636 10.3727/096368910X503415

[59] Blasi A , Martino C , Balducci L et al. Dermal fibroblasts display similar phenotypic and differentiation capacity to fat‐derived mesenchymal stem cells, but differ in anti‐inflammatory and angiogenic potential. Vasc Cell 2011;3:5. 21349162 10.1186/2045-824X-3-5 PMC3044104

[60] Molinos M , Almeida CR , Gonçalves RM et al. Improvement of bovine nucleus pulposus cells isolation leads to identification of three phenotypically distinct cell subpopulations. Tissue Eng Part A 2015;21:2216–2227. 25962534 10.1089/ten.TEA.2014.0461

[61] Risbud MV , Schoepflin ZR , Mwale F et al. Defining the phenotype of young healthy nucleus pulposus cells: Recommendations of the Spine Research Interest Group at the 2014 annual ORS meeting. J Orthop Res 2015;33:283–293. 25411088 10.1002/jor.22789 PMC4399824

