# Supplementary material for: Systemic Delivery of Bone Marrow Mesenchymal Stem Cells for In Situ Intervertebral Disc Regeneration
Source: Stem Cells Transl Med. 2016 Oct 11;6(3):1029–39. doi: 10.5966/sctm.2016-0033 (PMC5442789; doi:10.5966/sctm.2016-0033)
Supplement: Supplementary file 1 — Supporting Information [file SCT3-6-1029-s001.pdf]

Supplemental Information - Cunha et al.

Supplemental Figures

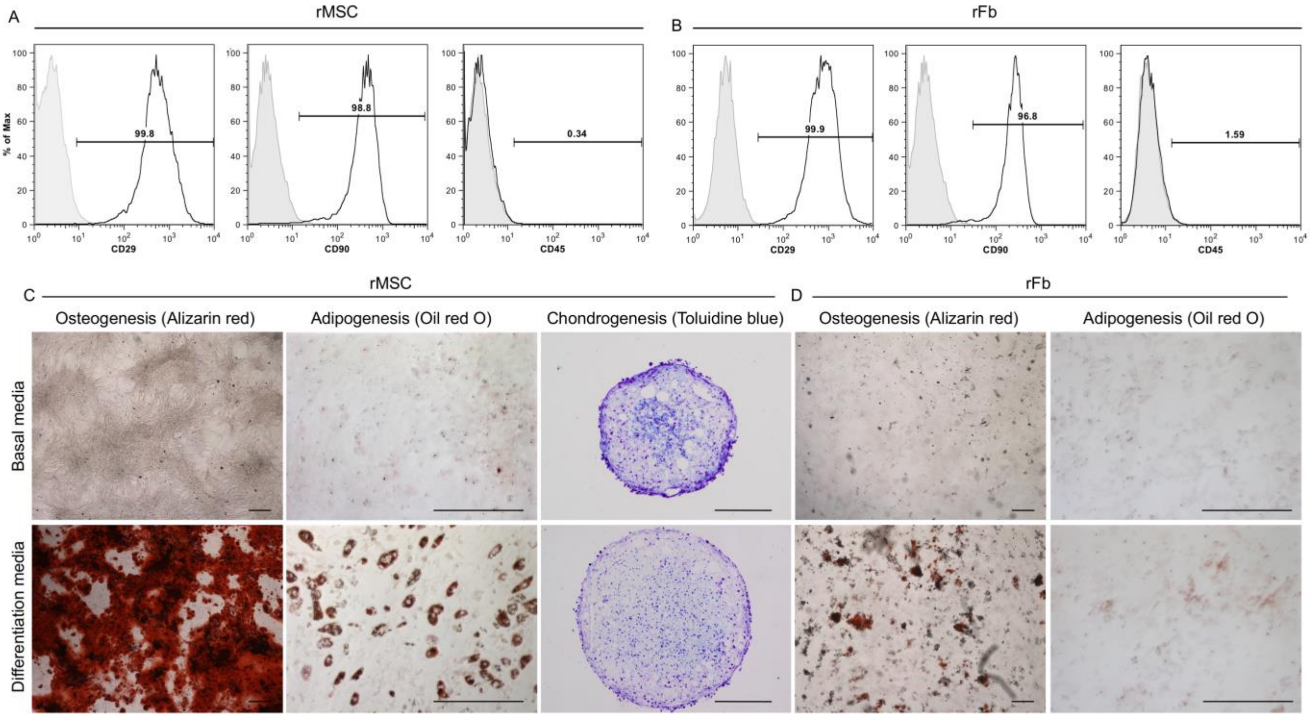

Figure S1. A, B) Phenotypic characterization of primary bone marrow MSCs. Representative histograms are shown in black line and the respective isotype controls are shown as grey. The following fluorochrome-labeled antibodies were used: CD29-Fluorescein isothiocyanate (FITC), CD90-Phycoerythrin (PE) and CD45-Allophycocyanin (APC). Data was collected on a FACSCantoTM II flow cytometer (Becton Dickinson) and analysis was performed with FlowJo software version 8.7. C,D) Differentiation assays assessed osteogenesis by Alizarin Red staining, adipogenesis by Oil red O staining and chondrogenesis by Toluidine staining, performed for rMSC (C) and rFb (D). rFb were unable to form chondrogenic pellets. Scale bars: 200 μm.

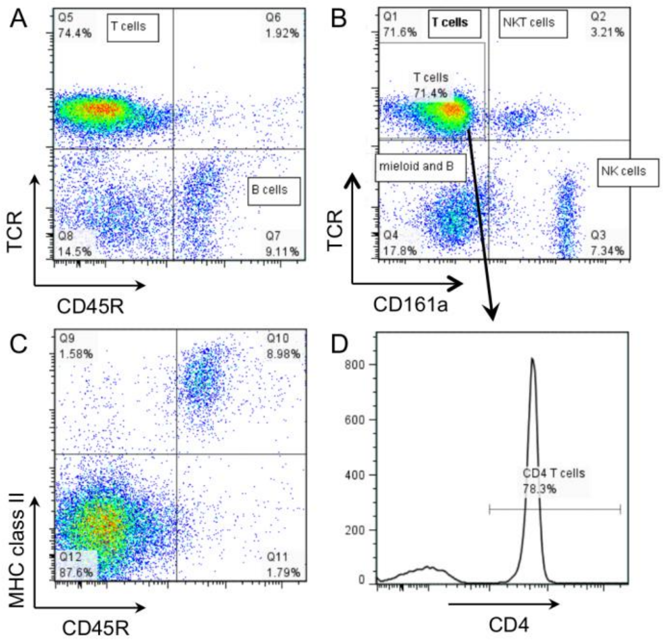

Figure S2. Representative flow cytometry dot plots showing discrimination of B- and T-cells (A), T-cells, NK, NKT and myeloid+B-cells (B), staining for MHCII+ cells within the CD45R population (C) and CD4+ cells within TCR+ population (D).

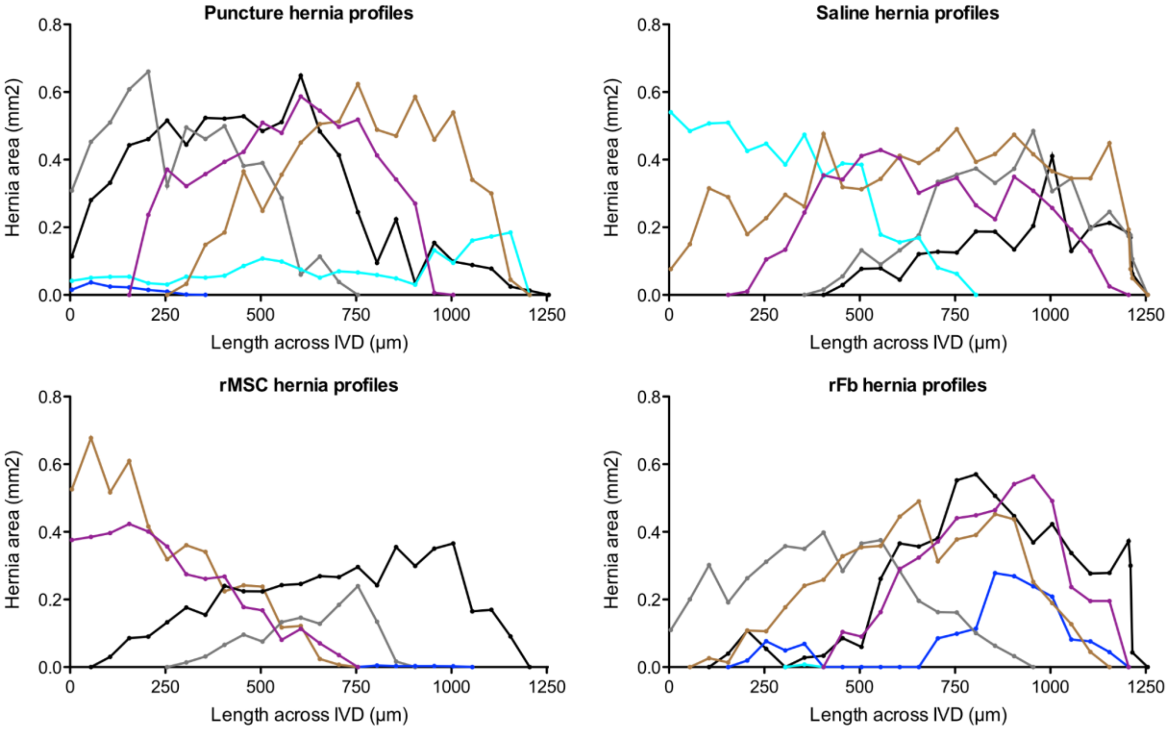

Figure S3. Hernia area profiles, quantified at precise intervals throughout the IVD. Tissue sections were stained for Safranin O & Fast Green and images of the hernia collected by an upright optical microscope. For each section, delimitation of the hernia was made by the freehand selection tool using the ImageJ software. After image scale calibration, the area of the selection in mm^2 was determined.

Supplemental Table

Table S1. Parameters used to calculate the histological grading score, each classified with a score between 1 (normal disc) and 5 (highly degenerated disc). Results presented as MEAN±SD.

Group	I. Cellularity AF	II. Morphology AF	III. Border NP/AF	IV. Cellularity NP	V. Morphology NP
naive	1.00±0.00	1.00±0.00	1.00±0.00	1.00±0.00	1.00±0.00
lesion	1.21±0.43	1.64±0.63	1.93±0.62	1.27±0.70	1.60±0.51
lesion+saline	1.13±0.35	1.40±0.51	2.07±0.80	1.17±0.58	1.75±0.75
lesion+rMSC	1.00±0.00	1.71±0.73	1.86±0.77	1.00±0.00	1.79±0.70
lesion+rFb	1.20±0.41	1.73±0.59	2.40±0.63	1.20±0.41	1.40±0.51
